# Impact of virtual monoenergetic images on the assessability of lower extremity arteries in (Poly-) trauma photon-counting detector CT

**DOI:** 10.1007/s10140-026-02502-1

**Published:** 2026-06-16

**Authors:** Daniel Dillinger, Christian Bauer, Hanns L. Kaatsch, Achim Neufang, Achim Hagen, Stephan Waldeck, Daniel Overhoff

**Affiliations:** 1 Department of Vascular and Endovascular Surgery, Federal Armed Services Hospital Koblenz, Ruebenacher Str. 170, Koblenz, 56072 Germany; 2 Department of Heart and Vascular Surgery, University Medical Center Johannes Gutenberg University Mainz, Mainz, Germany; 3 Department of Radiology and Neuroradiology, Federal Armed Services Hospital Koblenz, Koblenz, Germany; 4 Department of Neuroradiology, University Medical Centre Johannes Gutenberg University Mainz, Mainz, Germany; 5 Department of Radiology and Nuclear Medicine, University Medical Center Mannheim, Manheim, Germany

**Keywords:** Trauma, CT angiography, Photon counting detector CT

## Abstract

**Background:**

Computed tomography (CT) is state of the art imaging modality in patients after severe trauma. A double-bolus contrast media protocol can be used to combine both arterial and parenchymal contrast in one scan. In case of lower extremity trauma, the CT scans can be extended to include the legs in order to rule out vascular pathologies. Virtual monoenergetic (VME) images derived from a Photon-Counting detector CT (PCDCT) proved to be helpful in vascular diagnostics and could therefore also add information in these extended trauma scans. Aim of this study was to determine the VME reconstructions with sufficient vascular contrast (> 200 Hounsfield Units) and to examine, which VME level has best specificity and sensitivity as well as best subjective vascular visibility.

**Methods:**

In a retrospective analysis, we identified 34 patients undergoing extended whole-body trauma scans including lower extremities on a PCDCT with pathologies of the lower extremity arteries (extravasation, dissection etc.) and paired them with 34 patients without vascular pathologies. We measured CT values and noise of six vascular segments on energy levels 40 to 190 keV and calculated signal to noise (SNR) and contrast to noise (CNR) ratio. Subjective ratings for vascular visibility were performed on a five point Likert scale and two readers analyzed the images for vascular lesions across four energy levels (40, 70, 90 and 110 keV).

**Results:**

CT attenuation was measured above 200 HU below 88 keV in the common femoral artery, below 86 keV in the superficial femoral artery and below 85 keV in the popliteal artery. For the tibial arteries, we found higher CT values above 200 HU at the energy levels below 87 keV. Noise was highest at 40 keV for all energy levels, SNR and CNR for common femoral artery was highest at 40 keV.

Sensitivity peaked at 40 keV (78.4%) and showed decreasing values for higher energy levels. Specificity increased with higher keV levels up to 91.7%. 40 keV showed the highest sum of Likert ratings regarding vascular visibility.

**Conclusion:**

PCDCT derived VME reconstructions below 86 keV showed sufficient vascular contrast in extended whole-body trauma scans after double-bolus contrast media administration. Smaller vessel depiction can be hampered by increased noise on lower energy levels resulting in decreased image parameters.

Lower energy levels have the potential to improve the detection of vascular pathologies, such as bleeding and vascular occlusions.

## Introduction

Computed tomography (CT) plays a crucial role in patient evaluation in emergency settings. International guidelines emphasize the importance of CT examinations in trauma patients [1, 2]. In case of a suspected vascular trauma, CT angiographies are recommended by the European society of vascular surgery guidelines with a given specificity and sensitivity of 100% [3]. In trauma settings, patients typically undergo a non-contrast examination of the neurocranium, followed by a dedicated imaging protocol of the remaining body, including the pelvis [2]. These trauma scans can be used to assess patients with suspected vascular trauma (e.g. with extremity fractures/joint displacement, pre-hospitally suspected arterial bleeding) to plan the necessary endovascular or surgical approach by adding lower extremity runoff into the scan. Previous studies have demonstrated sufficient image quality for lower extremity runoff assessment in trauma CT [4]. In many cases, a double bolus contrast media protocol is used to combine both arterial and parenchymal contrast in one scan.

**Fig. 1:**
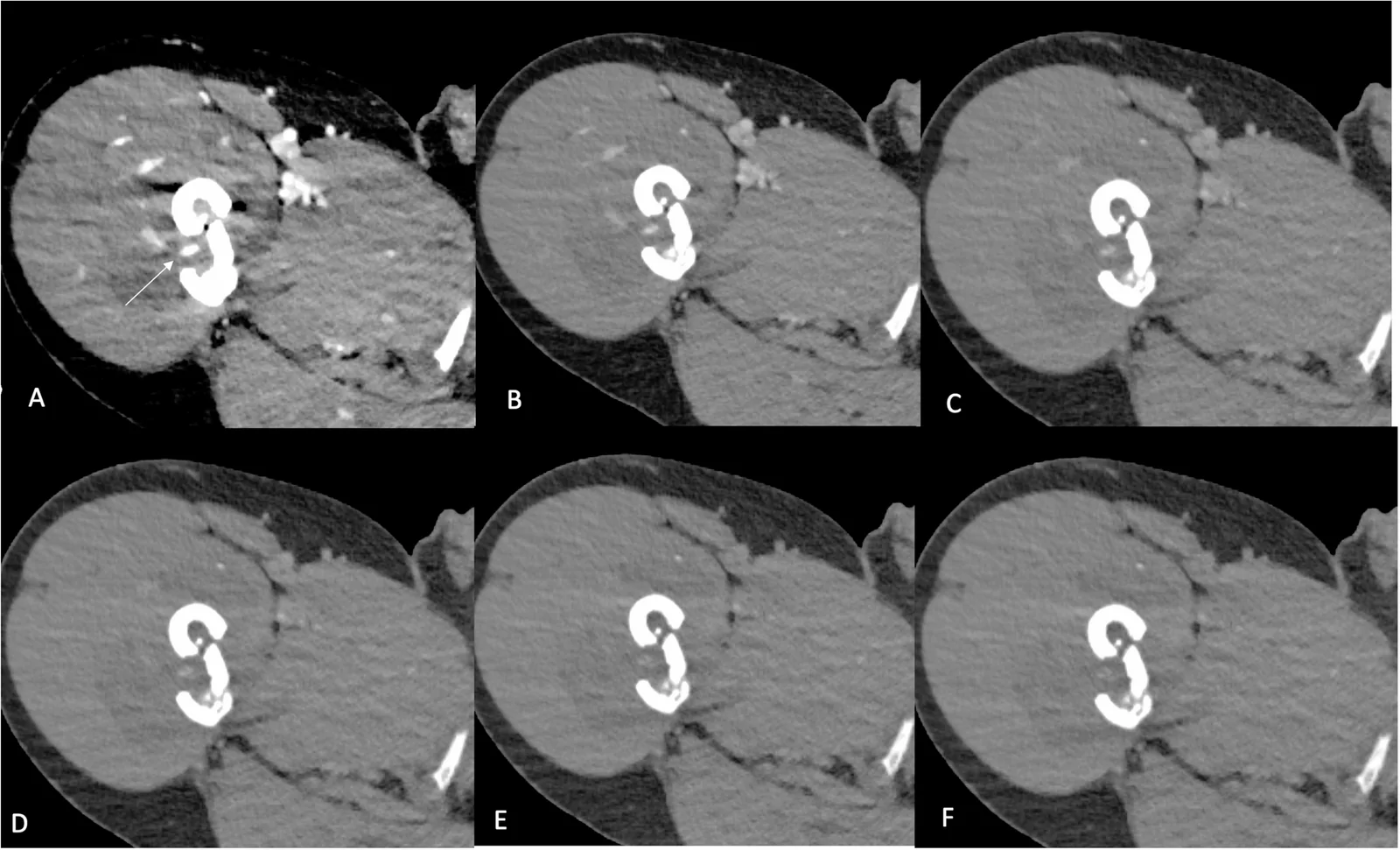
Demonstrates the effects of different energy levels on the reconstructions (A 40 keV, B 70 keV, C 90 keV, D 110 keV, E 150 keV, F 190 keV). Contrast media effects decrease with higher energy levels, the bleeding deriving from the femoral fracture (arrow) is most evident in the 40 keV reconstruction

Photon Counting Detector CT (PCDCT) offers less image noise with a reduced radiation dose and higher spatial resolution and allows virtual monoenergetic (VME) reconstructions on different energy levels [5, 6]. These reconstructions allow an increase of contrast media effects and can also be used for material decomposition analysis and have proved beneficial in many settings [7–10]. By increasing the contrast media effects on lower energy levels, vascular structures (and also contrast media extravasations) become more prominent [11]. This was previously described also for dual energy CT [12]. Figure 1 demonstrates the effects of VME reconstructions on a contrast media extravasation in a femoral fracture. This results in higher subjective vascular ratings but also showed higher noise and lower subjective overall image ratings [7]. Previous studies on dual energy CT scanners showed promising results for low-energy reconstructions with improved vascular assessability, in e.g. peripheral artery disease [13], so we were eager to explore the potential of low energy reconstructions derived from inherent spectral datasets of PCDCT for the assessment of vascular pathologies in trauma patients. To our knowledge, no research before has been executed on the diagnostic accuracy of different VME reconstructions on vascular lesions in whole-body trauma PCDCT scans in combination with image acquisition after double-bolus contrast media injection protocol.

## Materials and methods

### Study design

We retrospectively identified all patients that underwent a whole-body trauma CT scan with on the PCDCT in our institution from December 2021 to April 2025.

Among these patients, we selected the datasets, that included the lower extremities to at least the knee and identified patients with reported vascular pathology (contrast media stop, contour irregularities, relevant contrast media extravasation) of the lower extremities. We also identified the same number of patients without vascular pathology but relevant injuries of the legs as a control group. CT scans were excluded if the images and especially the vessels were hampered by major artifacts.

This retrospective analysis of PCD-CT data was approved by the local ethics committee. The need for written informed consent was waived owing to the retrospective nature of the study.

### Scanning protocol

All patients were examined with our dedicated trauma protocol on a PCDCT (NAEOTOM alpha Siemens Healthineers, Forchheim, Germany) that includes two topograms and modulates the tube current in z-axis with accordance to the patient’s diameter, attenuation was calculated by the acquired topograms.

Contrast medium (Xenetix 350; Guerbet, Roissy, France) was administered via an upper extremity vein using a power injector (CT Motion XD8000; Ulrich Medical, Ulm, Germany). In our trauma scans, a double bolus technique was used (60 ml contrast medium (flow 4 ml/s) followed by a 50 ml saline chaser of (flow 4 ml/s), after a pause of 15 s another 60 ml of contrast medium and a saline chaser of 50 ml with the same flow were injected.

The trauma CT scan was initiated using bolus tracking in the descending thoracic aorta with a predefined threshold of 100 HU and a delay of 70 s (CARE Bolus, Siemens Healthineers, Forchheim, Germany). Bolus tracking was used to scan the cervical vessels in an arterial phase, chest/abdomen/pelvic as well as lower extremity scans were then performed with the mixed bolus as described above. The scans were performed with CARE kV image quality level 145.

The data was acquired at 120 kV, and our scans were performed in a cranio-caudal direction with a pitch of 0.80 and an increment of 0.70 mm. Spectral post-processing (SPP) datasets were analyzed with a slice thickness of 1 mm in a Qr40 kernel.

We also obtained the dose length product (DLP) and CT dose index (CTDIvol) and as a replacement for BMI, we calculated the effective patient diameter (D_eff_) according to O’Neil et al. [14]

### Objective analysis

We used Syngo.via (VB60, Siemens Healthineers, Forchheim, Germany) software to place two regions of interest (ROI) at the VME level of 70 keV in the common femoral artery on both sides, in the superficial femoral artery as well as in the popliteal artery in Monoenergetic+ mode. We also placed one ROI in each of the tibial arteries on both sides. Afterwards, attenuation and standard deviation (noise) was calculated on all energy levels between 40 keV and 190 keV in 1 keV increments. Attenuation was grouped into more than 200 Hounsfield Units (HU), between 150 and 199 HU and below 150 HU as described previously for trauma scans [4, 15]. We additionally calculated signal-to-noise ratio (SNR) and contrast-to-noise ratio (CNR) based on equations, which were also previously published for lower extremity imaging [16].

### Subjective analysis and diagnostic accuracy

For subjective analysis, we reconstructed the datasets on 40, 70, 90 and 110 keV at 1 mm slice thickness with a transversal orientation from slightly above the aortic bifurcation down to the distal end of the scan range. These reconstructions were pseudonymized and presented to two readers (both board-certified radiologists with seven and six years of experience in trauma imaging) in a randomized order. Both readers were therefore blinded to the patient data, to the reconstructed energy level as well as to the fact, if the initial report of the scan contained the diagnosis of a vascular pathology. Readers were allowed to change the window settings and perform multiplanar reconstructions and judge the images regarding a visible vascular pathology and as well on a subjective scale for vascular visibility which is depicted in the Table 1.

**Table 1:** Visualizes the subjective Likert scale for vascular visibility

Likert Rating	Vascular visibility
1	severe noise and artifacts, unacceptable image quality
2	high noise or heavy artifacts; unclear vessel contours, poor image quality
3	mild noise or artifacts; partially blurred vessel contours, average image quality
4	low noise or artifacts; clear vessel contours, good image quality
5	minimal noise and artifacts, clear vessel contours, excellent image quality

In addition, when both observers identified a vascular injury that had not been mentioned in the initial report, the original images were re-evaluated by a third radiologist with 15 years of experience in emergency radiology to determine whether a vascular pathology had already been present but not reported. Sensitivity and specificity calculations were again performed with this new dataset.

### Statistical analysis

Statistical analysis was performed with SPSS 30 (IBM, Armonk, NY, USA). Kolmogorov-Smirnov test was used to test for normal distribution. Estimates were given as averages ± standard deviation if normally distributed, otherwise median (quartile 1; quartile 3) or frequency (percentage) were used. To calculate specificity and sensitivity we used crosstables.

We calculated Cohen’s kappa for inter-reader correlation and considered > 0.81 almost perfect, 0.61–0.80: substantial, 0.41–0.60 was considered as moderate, 0.21–0.4 fair and less than 0.2 slight, below 0 was considered poor.

## Results

### Study population

Out of 1167 trauma scans in the given time frame, 1013 were performed on the PCDCT. 121 included lower extremity scans. Out of these, we identified 34 patients with vascular pathologies of the lower extremities and therefore paired them with 34 other patients with injuries of the lower extremities visible in the CT scans but without vascular pathology.

The following Table 2 shows the dose data and other study population data.

**Table 2:** Demonstrates the population data overall and grouped for vascular and no vascular pathology within the scan

	Overall	Vascular pathology	No vascular pathology
Male	47/68 (69.1%)	25/34 (73.5%)	22/34 (64.7%)
Female	21/68 (30.9%)	9/34 (26.5%)	12/34 (35.3%)
Age (years)	49±23	54±23	44±22
D_eff_ (cm)	28.8±4.9	29.3±5.6	28.4±4.2
CTDivol (mGy)	7.1±2.1	6.8±2.2	7.4±1.9
DLP (mGycm)	976±269	971±299	981±239

### Objective image parameters

We measured 272 out of 272 vascular segments of the common femoral artery (100%), 262 of 272 (96%) of the superficial femoral artery and 248 of 272 (91%) of the popliteal artery. For the tibial arteries (because of three possible ROIs on each side), we measured 332 out of 408 segments (81%). Reasons for lacking vascular segment measurements were no visible contrast media because of peripheral artery disease, attached tourniquets, vascular lesions or CT scans which did not include the specific segment.

CT values decreased with increasing energy levels. Overall, the common femoral artery demonstrated highest attenuation values and the arteries of the calve had lowest attenuation values (e.g. at 40 keV for the common femoral artery median of 898 HU vs. tibial arteries 696 HU). Common femoral artery attenuation decreased below 200 HU between 88 and 89 keV and went below 150 HU from 104 to 105 keV. For the superficial femoral artery, we found values below 200 HU above the energy of 86 keV and below 150 HU above the levels of 101 keV.

Popliteal artery showed lower attenuation than 200 HU above 85 keV and lower values than 150 HU above 101 keV. For the tibial arteries, we found lower CT values than 200 HU at the energy levels above 87 keV and lower values than 150 HU at the energy levels of 92 keV.

**Fig. 2:**
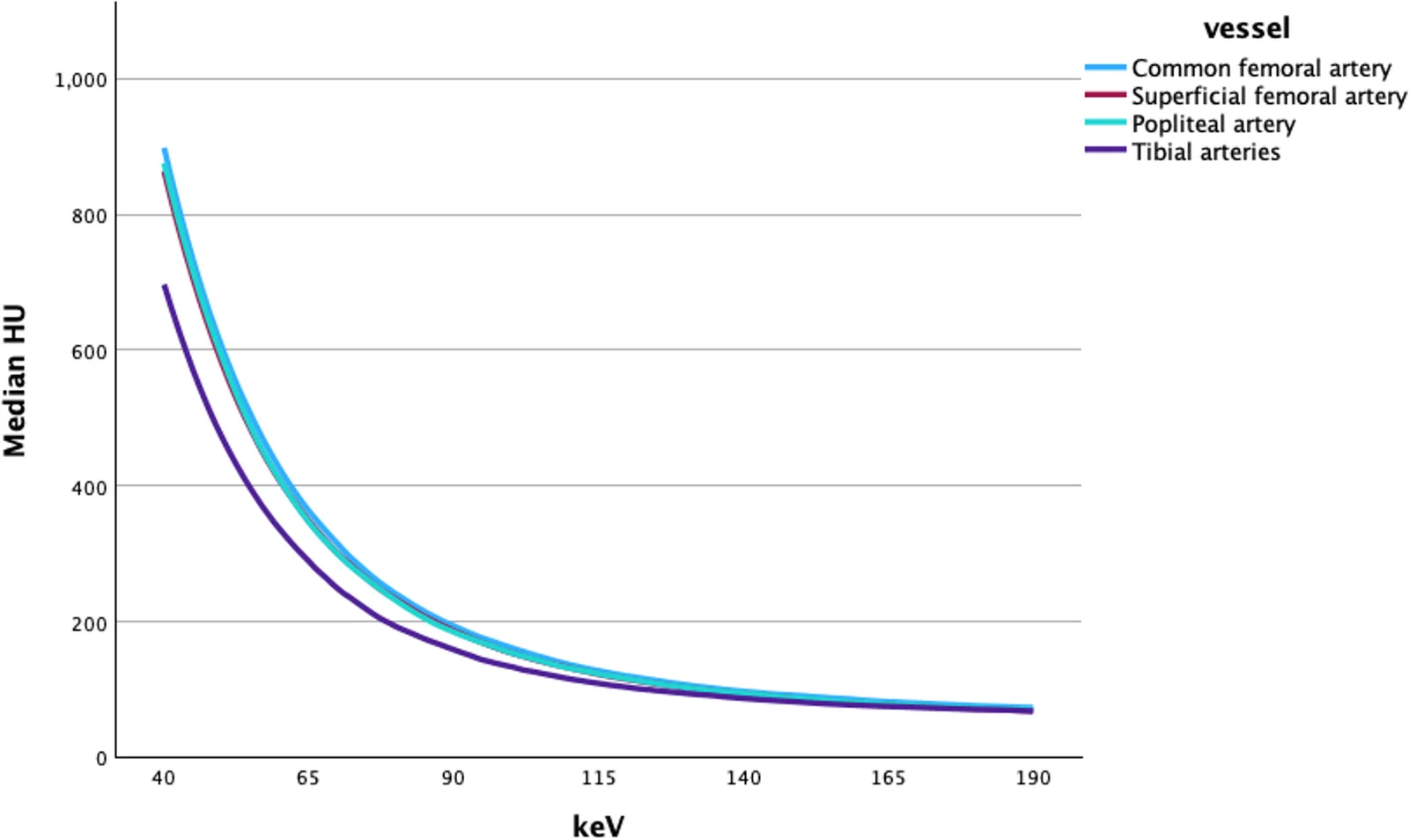
Demonstrates the median CT values of the lower extremity vascular segments on the different energy levels

The medians of the vascular segment CT values are shown in Fig. 2.

**Fig. 3:**
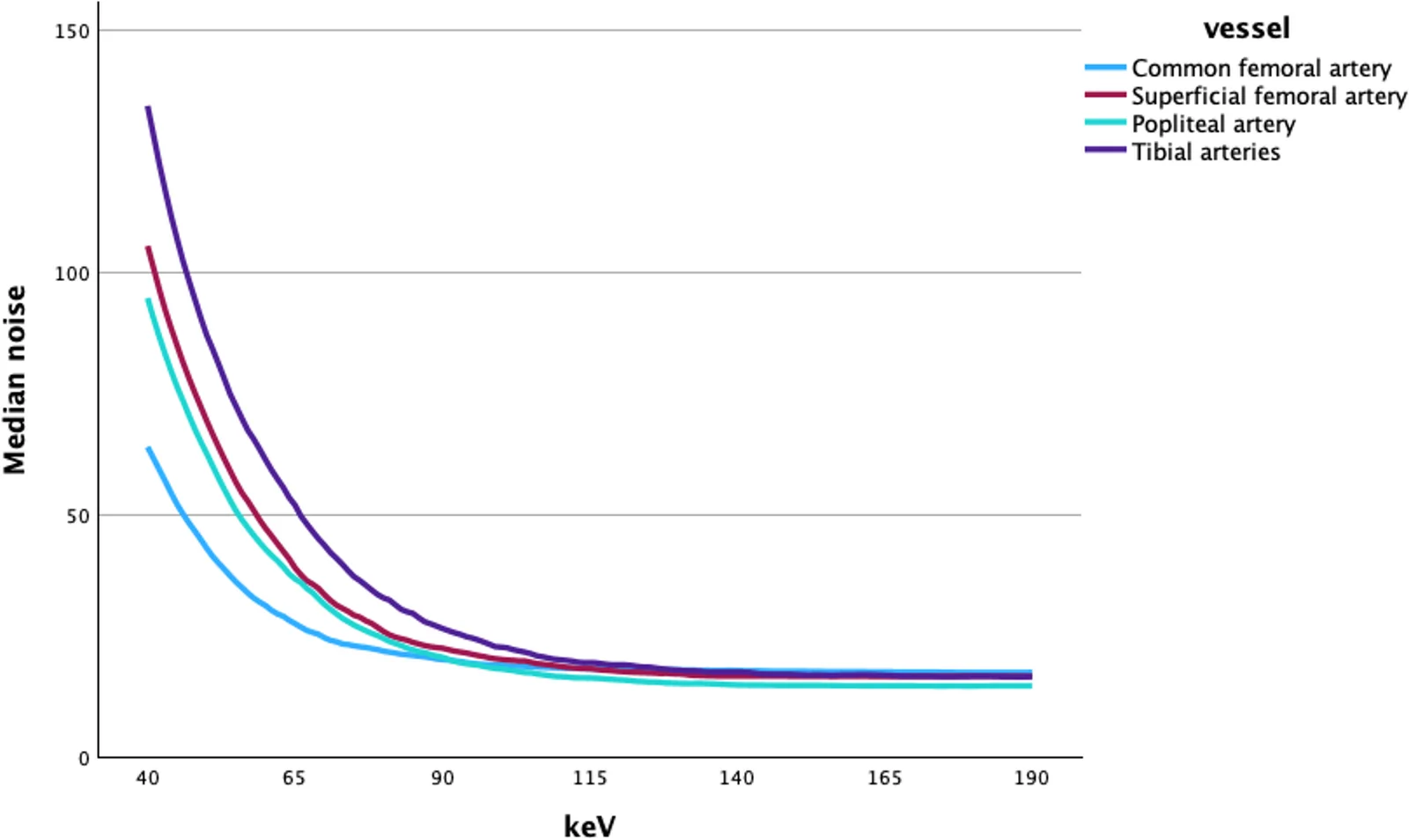
Shows the noise levels of the vascular segments, depending from the energy level

Noise values were most prominent in tibial arteries as well, lowest noise values were seen in the common femoral artery (e.g. for 40 keV a median of 64 for the common femoral artery and 134 for the tibial arteries). Noise decreased with higher energy levels (at 70 keV common femoral artery showed a median noise of 25 and tibial arteries of 44). The following figure shows the different noise values of the vascular segments. The noise of the popliteal artery is lowest on higher energy levels than the other vascular segments (Fig. 3).

**Fig. 4:**
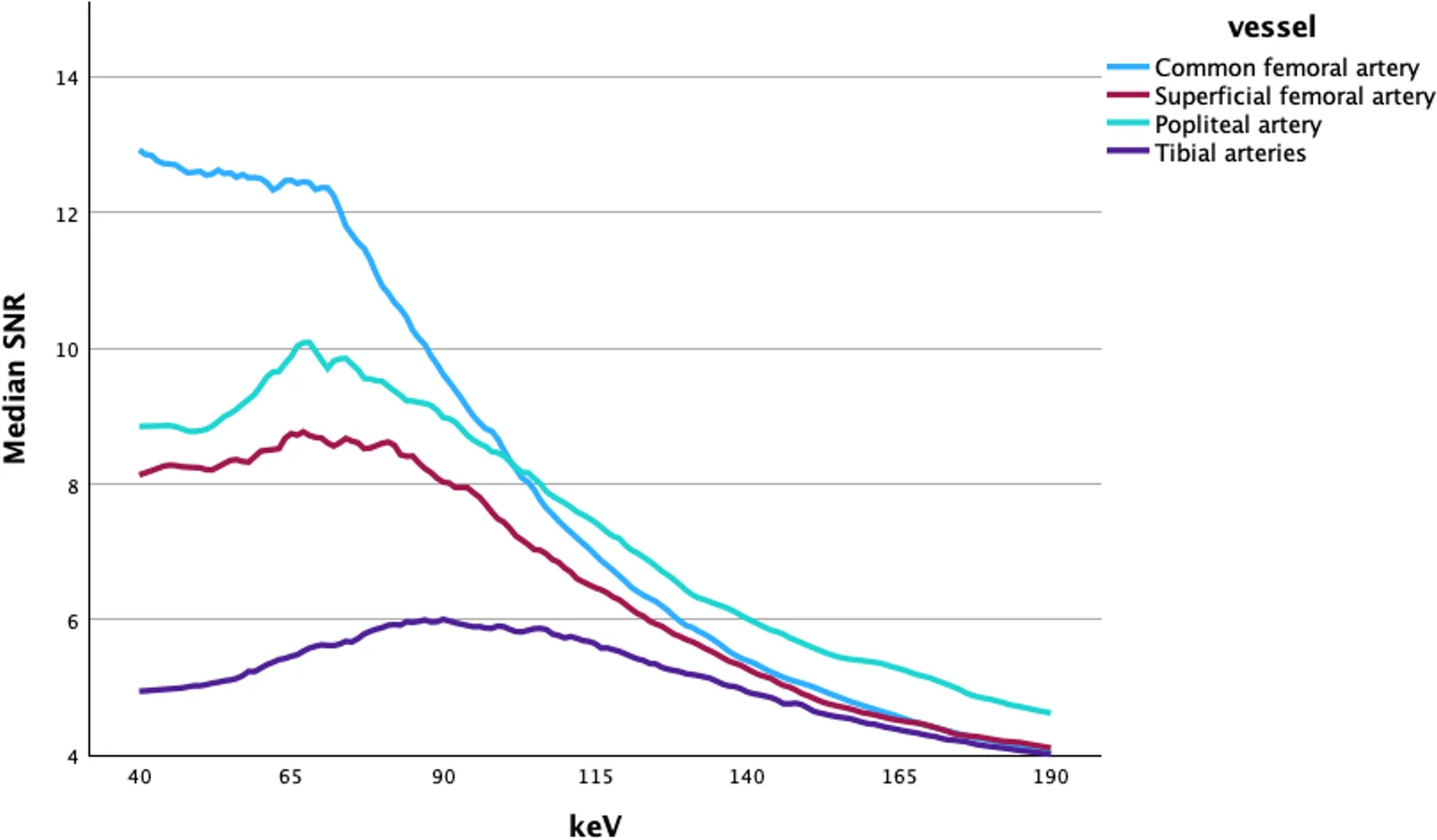
Demonstrates the CNR of the vascular segments on the dedicated energy level

As shown in Fig. 4 CNR shows a steady decrease with increasing energy level with a small plateau at 63–65 keV for all vascular segments. At the highest energy level (190 keV), CNR was nearly uniform across all segments, whereas at lower energy levels, differences between vascular segments became more pronounced.

**Fig. 5:**
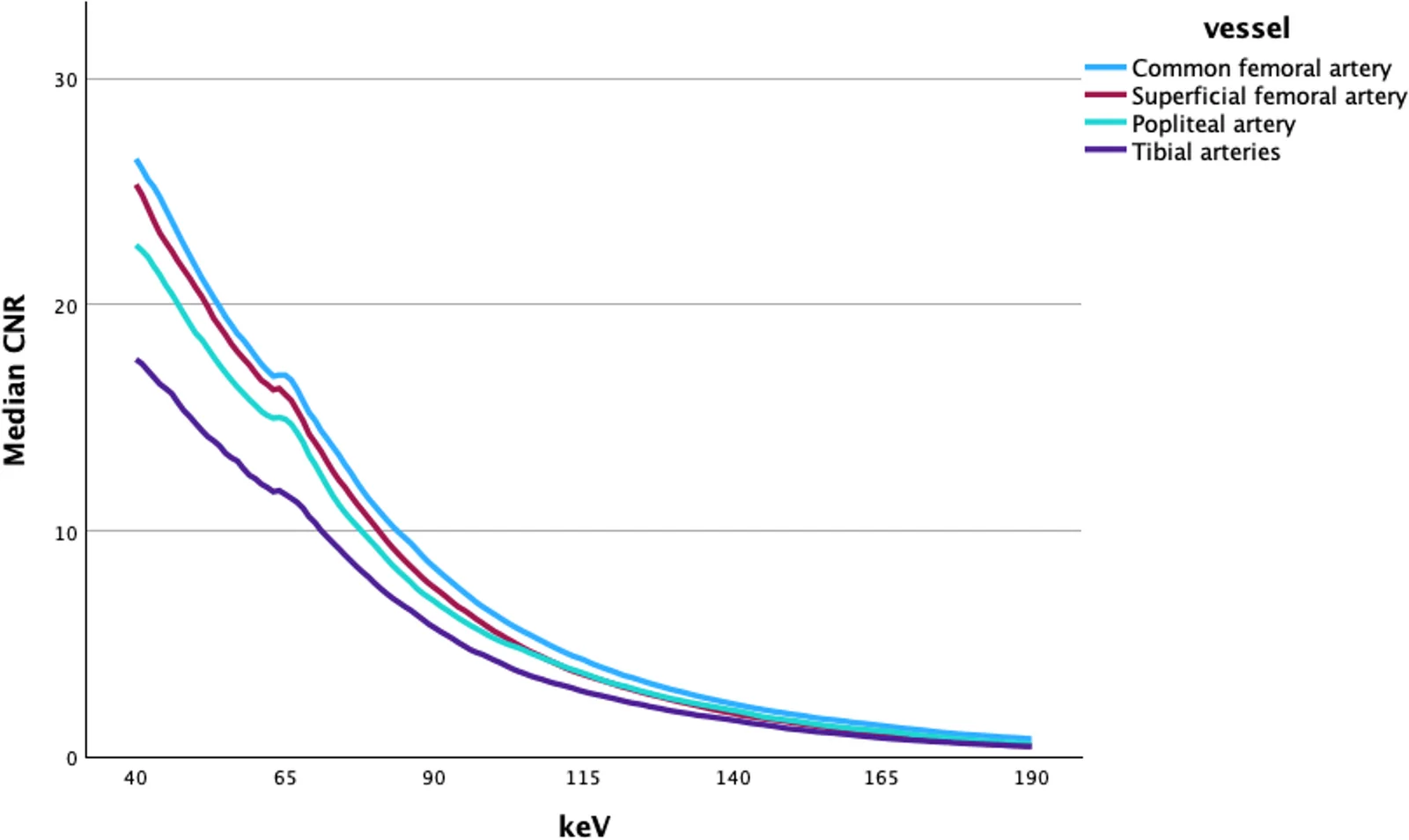
Shows the different SNR of the vascular segments on the different energy levels

The popliteal artery had highest SNR at the higher energy levels, which corresponds with the lowest noise on these reconstructions. All vascular segments showed a steady decline of SNR for higher energy levels which is shown in Fig. 5. SNR for the common femoral artery showed highest values for the lowest energy levels, but superficial femoral artery and popliteal artery have a slight increase up to e.g. 10 for the popliteal artery on the level of 66 to 68 keV. These levels also include the peak of the superficial femoral artery. Tibial arteries showed their maximum SNR at 90 keV (median of 6).

### Subjective image parameters

**Fig. 6:**
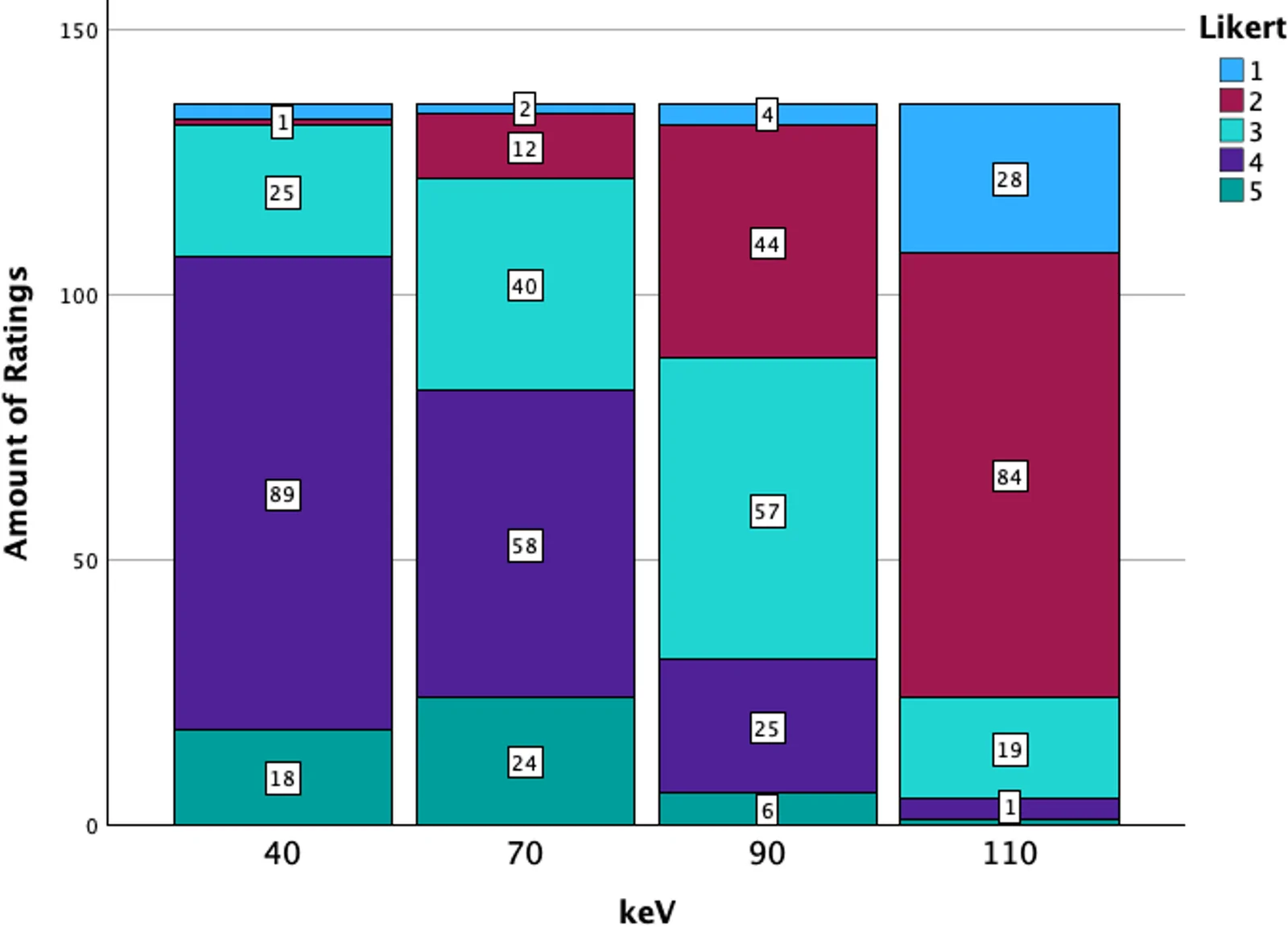
Demonstrates the amount of the different Likert ratings regarding the vascular assessment

As shown in Figs. 6 and 40 keV showed predominantly ratings of 4 and 5, 70 keV has slightly more top ratings, but less ratings of “4”. The sum of all ratings at 40 keV was 526, at 70 keV 486, at 90 keV 384 and at 110 keV 270. The other two energy levels showed decreasing subjective ratings in our study, this means, that the lower energy levels received overall higher ratings than the two higher energy levels examined in our study with increasing ratings of even non-diagnostic scans on 90 keV and even more on 110 keV. Interreader-correlation was fair with 0.29.

### Sensitivity and specificity on different energy levels

As energy levels increased, a reduction in the overall sensitivity for detecting vascular trauma was observed, whereas specificity showed a gradual increase at higher energy levels. The highest sensitivity for vascular trauma detection was measured at 40 keV with 78.4%, declining to 59.2% at 110 keV. In contrast, specificity demonstrated a slight increase with rising energy levels, from 82.3% at 40 keV to 91.7% at 110 keV.

The following (Tables 3 and 4) present the sensitivity and specificity based on the original reports and after inclusion of newly identified, previously unreported vascular lesions.

**Table 3:** Demonstrates overall sensitivity and specificity as well as interobserver variation range depending on different energy levels before manual correction of questionable cases

keV	Sensitivity in total	Specificity in total	Interobserver variation on sensitivity	Interobserver variation on specificity
40	77.9%	76.5%	64.7–91.2	64.7–88.2
70	76.5%	83.8%	67.6–85.3	79.4–88.2
90	72.1%	88.2%	70.6–73.5	82.4–94.1
110	61.8%	88.2%	55.9–67.6	88.2

**Table 4:** Demonstrates overall sensitivity and specificity as well as interobserver variation range depending on different energy levels after manual correction of questionable cases

keV	Sensitivity in total	Specificity in total	Interobserver variation on sensitivity	Interobserver variation on specificity
40	78.4%	82.2%	67.6–89.2	67.7–96.8
70	73%	85.5%	69.4–81.1	80.6–90.3
90	68.9%	90.3%	67.6–70.3	83.9–96.8
110	59.2%	91.7%	55.3–63.2	90-93.3

## Discussion

The aim of this study was to assess the diagnostic quality of lower extremity arteries in trauma CT scans using VME reconstructions. To achieve this, we analyzed lower extremity vessels across energy levels ranging from 40 to 190 keV and observed a decrease in CNR with increasing energy levels. Furthermore, we evaluated diagnostic parameters at 40, 70, 90, and 110 keV. Sensitivity declined at higher energy levels, whereas lower keV values enabled the detection of additional vascular pathologies that had not been initially reported.

In terms of diagnostic attenuation, VME reconstructions below the energy level of 85 keV showed sufficient attenuation values for every vascular segment we examined. Overall, differences between vascular segments regarding their cutoff for sufficient attenuation were small (ranging between 85 keV and 88 keV). The increased noise in smaller vessels is in line with previous research [6]. The differences in attenuation of the vascular segments might be caused by contrast media flow, further dilution effects and decreased cardiac output in some of the patients. This also leads to the discrepancies in CNR. Highest CNR for lowest energy level was already described for many other vascular territories, e.g. carotid arteries by Booz et al. [17].

Increased noise in lower VME-reconstructions of smaller vessels explains discrepancies of their SNR compared to more proximal vessels. The SNR of larger vessels thus follows previous studies [17]. Jia et al. already performed research on 55 keV reconstructions in lower extremity angiographies [18] and found higher CNR and SNR compared to standard reconstructions in the VME group also for below the knee segments. In their results, a decrease in attenuation from larger to smaller vessels can be seen. But differently from our research, they only observed one energy level and used a dedicated angiography contrast media protocol, not a mixed one for trauma scans like we did.

The observed CNR plateau around 63–65 keV may be attributed to the specifics of the employed algorithm for the calculation of the virtual monoenergetic images, as well as the keV-dependent iodine contrast and noise behavior, in particular in the legs. The legs are an area of rather low attenuation. Low attenuation is associated with lower image noise at constant radiation dose, and at the same time it means a lower effective energy of the detected spectrum and thus a higher intrinsic iodine contrast.

Previous studies used VME reconstructions also to decrease metal artifacts, their optimum level ranging from 110 keV to 130 keV depending on the indication/the metal implant the artifact is originating from [19–23]. Given the fact of CT attenuation decreasing below 200 HU even at lower energy levels (even below 90 keV) in our study, this would support the conclusion, that solely using VME reconstructions for metal artifact reduction would lead to an insufficient vascular contrast and underlines the importance of metal artifact reconstruction algorithms additionally, which is also in line with previous research [24, 25].

The present study demonstrates the impact of different energy levels on the visualization of vascular pathologies. Sensitivity for the detection of vascular injury was highest at 40 keV and progressively declined with increasing energy levels up to 110 keV, decreasing from 78.4% to 59.2%. In contrast, specificity showed a slight increase with higher energy levels, rising from 82.3% at 40 keV to 91.7% at 110 keV. Notably, a considerable interobserver variation was observed, with sensitivity ranging from 67.6% to 89.2% and specificity from 67.7% to 96.8% at the same energy level. These findings are consistent with previous studies demonstrating improved SNR and CNR with virtual monoenergetic reconstructions [16–18] and highlighting the advantages of dual-energy CT for the visualization of vascular trauma [26]. Using lower energy levels led to the identification of more vascular trauma patients, which resulted in higher sensitivity and specificity levels at the energy level of 40 keV (Tables 3 and 4).

This study has several limitations. First is the retrospective monocentric study design. Secondly, the observed number of patients is low, a greater number would increase the power of the research and allow subgroup analysis (which we could not perform due to the low number of patients). Another point is the heterogeneity of the population, trauma patients vary in age, previous diseases etc. and this also influences the current study (especially different cardiac outputs could make a big impact on contrast media dynamics with bigger impacts especially for the lower extremities). Also, due to the retrospective design, not all clinical data was available, so the medical history of the patients was not taken into account. Furthermore, the study design, in which identical trauma and control cases were reviewed at multiple energy levels, may have introduced observer bias. Recognition of previously evaluated cases could have allowed observers to recall whether a vascular injury had been identified on another, potentially more sensitive energy level (e.g., 40–70 keV), thereby influencing their interpretation.

## Conclusion

Based on the results of that retrospective study with a limited sample size, virtual monoenergetic reconstructions significantly influence visualization of lower extremity arteries in trauma CT. Lower keV levels provide higher attenuation and improved CNR, resulting in greater sensitivity for detecting vascular injuries. Adequate vascular contrast was maintained below approximately 85 keV with only minor segmental differences. Higher keV levels reduced sensitivity but may still be useful for metal artifact reduction when combined with dedicated algorithms. Low-keV reconstructions, particularly around 40–70 keV, may therefore improve detection of clinically relevant vascular injuries in trauma imaging.

## References

[1] Benning D, Hackenberg L, Pavlu F, Weber W, Franke A, Kollig E et al (2024) New Recommendations for the Care of Severely Injured Patients: Revision of the S3 Guideline on Treatment of Polytrauma/Severe Injuries. Z Orthop Unfall 162(6):630–637 10.1055/a-2276-6357 38810965

[2] Valcarcel CR, Bieler D, Bass GA, Gaarder C, Hildebrand F, Group EPC (2025) ESTES recommendations for the treatment of polytrauma-a European consensus based on the German S3 guidelines for the treatment of patients with severe/multiple injuries. Eur J Trauma Emerg Surg 51(1):171 10.1007/s00068-025-02852-4 PMC11991986 40214785

[3] Wahlgren CM, Aylwin C, Davenport RA, Davidovic LB, DuBose JJ, Gaarder C et al (2025) Editor's choice -- European Society for Vascular Surgery (ESVS) 2025 clinical practice guidelines on the management of vascular trauma. Eur J Vasc Endovasc Surg 69(2):179–237 10.1016/j.ejvs.2024.12.018 39809666

[4] Foster BR, Anderson SW, Uyeda JW, Brooks JG, Soto JA (2011) Integration of 64-detector lower extremity CT angiography into whole-body trauma imaging: feasibility and early experience. Radiology 261(3):787–795 10.1148/radiol.11100604 21940506

[5] Becker BV, Kaatsch HL, Nestler K, Overhoff D, Schneider J, Dillinger D et al (2023) Initial experience on abdominal photon-counting computed tomography in clinical routine: general image quality and dose exposure. Eur Radiol 33(4):2461–2468 10.1007/s00330-022-09278-1 PMC10017564 36477938

[6] Dillinger D, Overhoff D, Booz C, Kaatsch HL, Piechotka J, Hagen A et al (2023) Impact of CT Photon-counting virtual monoenergetic imaging on visualization of abdominal arterial vessels. Diagnostics (Basel) 13(5) 10.3390/diagnostics13050938 PMC10000913 36900082

[7] Dillinger D, Overhoff D, Ayx I, Kaatsch HL, Hagen A, Schonberg SO et al (2024) Optimizing arterial vessel contrast in portal venous phase with virtual monoenergetic images from photon-counting detector CT scans of the abdomen-first clinical experiences. Diagnostics (Basel) 14(6) 10.3390/diagnostics14060627 PMC10969743 38535047

[8] Dillinger D, Overhoff D, Froelich MF, Kaatsch HL, Booz C, Hagen A et al (2024) Photon-counting detector CT virtual monoenergetic images in cervical trauma imaging-optimization of dental metal artifacts and image quality. Diagnostics (Basel) 14(6) 10.3390/diagnostics14060626 PMC10968735 38535045

[9] Dillinger D, Waldeck S, Overhoff D, Faby S, Jurgens M, Schmidt B et al (2025) Automated Kidney Stone Composition Analysis with Photon-Counting Detector CT, a Performance Study-A Phantom Study. Acad Radiol 32(4):2005–2012 10.1016/j.acra.2024.10.045 39550346

[10] Kaatsch HL, Vollmecke MF, Becker BV, Dillinger D, Kubitscheck L, Wohler A et al (2024) Improved Discriminability of Severe Lung Injury and Atelectasis in Thoracic Trauma at Low keV Virtual Monoenergetic Images from Photon-Counting Detector CT. Diagnostics (Basel) 14:19 10.3390/diagnostics14192231 PMC11475707 39410635

[11] Euler A, Higashigaito K, Mergen V, Sartoretti T, Zanini B, Schmidt B et al (2022) High-Pitch Photon-Counting Detector Computed Tomography Angiography of the Aorta: Intraindividual Comparison to Energy-Integrating Detector Computed Tomography at Equal Radiation Dose. Invest Radiol 57(2):115–121 10.1097/RLI.0000000000000816 34352805

[12] Albrecht MH, Vogl TJ, Martin SS, Nance JW, Duguay TM, Wichmann JL et al (2019) Review of Clinical Applications for Virtual Monoenergetic Dual-Energy CT. Radiology 293(2):260–271 10.1148/radiol.2019182297 31502938

[13] Fahrni G, Mingas T, Deliessche A, Hraichi S, Rotzinger DC, Si-Mohamed SA et al (2023) Low-iodine 40-keV virtual monoenergetic CT angiography of the lower extremities. Front Cardiovasc Med 10:1276738 10.3389/fcvm.2023.1276738 PMC10629994 37942065

[14] O’Neill S, Kavanagh RG, Carey BW, Moore N, Maher M, O’Connor OJ (2018) Using body mass index to estimate individualised patient radiation dose in abdominal computed tomography. Eur Radiol Exp 2(1):38 10.1186/s41747-018-0070-5 PMC6258803 30483977

[15] Anderson SW, Foster BR, Soto JA (2008) Upper extremity CT angiography in penetrating trauma: use of 64-section multidetector CT. Radiology 249(3):1064–1073 10.1148/radiol.2493080652 19011196

[16] Mei W, Tang Q, Li C, Wang S, Zhou Q, Xu L et al (2025) Improving lower-extremity artery depiction and diagnostic confidence using dual-energy technique and popliteal artery monitoring in dual-low dose CT angiography. Sci Rep 15(1):16143 10.1038/s41598-025-97868-6 PMC12062275 40341131

[17] Booz C, Bucolo GM, D’Angelo T, Mazziotti S, Lanzafame LRM, Yel I et al (2024) Carotid artery assessment in dual-source photon-counting CT: impact of low-energy virtual monoenergetic imaging on image quality, vascular contrast and diagnostic assessability. Radiol Med 129(11):1633–1643 10.1007/s11547-024-01889-6 PMC11554704 39287697

[18] Jia X, Li X, Li J, Tian Q, Yao Y, Zhu S et al (2020) Improving diagnostic accuracy for arteries of lower extremities with dual-energy spectral CT imaging. Eur J Radiol 128:109061 10.1016/j.ejrad.2020.109061 32442836

[19] Marth AA, Goller SS, Kajdi GW, Marcus RP, Sutter R, Photon-Counting Detector CT (2024) Clinical Utility of Virtual Monoenergetic Imaging Combined With Tin Prefiltration to Reduce Metal Artifacts in the Postoperative Ankle. Invest Radiol 59(8):545–553 10.1097/RLI.0000000000001058 38214560

[20] Patzer TS, Grunz JP, Huflage H, Hennes JL, Pannenbecker P, Gruschwitz P et al (2024) Ultra-high resolution photon-counting CT with tin prefiltration for bone-metal interface visualization. Eur J Radiol 170:111209 10.1016/j.ejrad.2023.111209 37992609

[21] Popp D, Sinzinger AX, Decker JA, Braun F, Bette S, Risch F et al (2023) Spectral metal artifact reduction after posterior spinal fixation in photon-counting detector CT datasets. Eur J Radiol 165:110946 10.1016/j.ejrad.2023.110946 37399668

[22] Rau A, Straehle J, Stein T, Diallo T, Rau S, Faby S et al (2023) Photon-Counting Computed Tomography (PC-CT) of the spine: impact on diagnostic confidence and radiation dose. Eur Radiol 33(8):5578–5586 10.1007/s00330-023-09511-5 PMC10326119 36890304

[23] Schreck J, Laukamp KR, Niehoff JH, Michael AE, Boriesosdick J, Woltjen MM et al (2023) Metal artifact reduction in patients with total hip replacements: evaluation of clinical photon counting CT using virtual monoenergetic images. Eur Radiol 33(12):9286–9295 10.1007/s00330-023-09879-4 PMC10667386 37436505

[24] Patzer TS, Kunz AS, Huflage H, Gruschwitz P, Pannenbecker P, Afat S et al (2023) Combining virtual monoenergetic imaging and iterative metal artifact reduction in first-generation photon-counting computed tomography of patients with dental implants. Eur Radiol 33(11):7818–7829 10.1007/s00330-023-09790-y PMC10598126 37284870

[25] Risch F, Decker JA, Popp D, Sinzinger A, Braun F, Bette S et al (2023) Artifact Reduction From Dental Material in Photon-Counting Detector Computed Tomography Data Sets Based on High-keV Monoenergetic Imaging and Iterative Metal Artifact Reduction Reconstructions-Can We Combine the Best of Two Worlds? Invest Radiol 58(9):691–696 10.1097/RLI.0000000000000967 36897803

[26] Joshi R, LeBedis C, Dao K, Qureshi M, Gupta A (2022) Dual energy CT angiography for lower extremity trauma: comparison with conventional CT. Emerg Radiol 29(3):471–477 10.1007/s10140-022-02037-1 35246779

